# Small-Molecules Selectively Modulate Iron-Deficiency Signaling Networks in Arabidopsis

**DOI:** 10.3389/fpls.2019.00008

**Published:** 2019-01-31

**Authors:** Sakthivel Kailasam, Wei-Fu Chien, Kuo-Chen Yeh

**Affiliations:** Agricultural Biotechnology Research Center, Academia Sinica, Taipei, Taiwan

**Keywords:** *Arabidopsis thaliana*, chemical biology, iron deficiency signaling, iron homeostasis, small-molecules

## Abstract

Plant growth requires optimal levels of iron (Fe). Fe is used for energy production, numerous enzymatic processes, and is indispensable for cellular metabolism. Recent studies have established the mechanism involved in Fe uptake and transport. However, our knowledge of Fe sensing and signaling is limited. Dissecting Fe signaling may be useful for crop improvement by Fe fortification. Here, we report two small-molecules, R3 and R6 [where R denotes repressor of *IRON-REGULATED TRANSPORTER 1 (IRT1)*], identified through a chemical screening, whose use blocked activation of the Fe-deficiency response in *Arabidopsis thaliana*. Physiological analysis of plants treated with R3 and R6 showed that these small molecules drastically attenuated the plant response to Fe starvation. Small-molecule treatment caused severe chlorosis and strongly reduced chlorophyll levels in plants. Fe content in shoots was decreased considerably by small-molecule treatments especially in Fe deficiency. Small-molecule treatments attenuated the Fe-deficiency-induced expression of the Fe uptake gene *IRT1*. Analysis of FER-LIKE IRON-DEFICIENCY-INDUCED TRANSCRIPTION FACTOR (FIT) and subgroup Ib *basic helix-loop-helix* (*bHLH*) gene (*bHLH38/39/100/101*) expression showed that R3 affects the FIT-network, whereas R6 affects both the FIT and Ib bHLH networks. An assessment of the effects of the structural analogs of R3 and R6 on the induction of Fe-dependent chlorosis revealed the functional motif of the investigated chemicals. Our findings suggest that small-molecules selectively modulate the distinct signaling routes that operate in response to Fe-deficiency. R3 and R6 likely interrupt the activity of key upstream signaling regulators whose activities are required for the activation of the Fe-starvation transcriptional cascade in Arabidopsis roots.

## Introduction

Many cellular functions occurring during plant growth and development depend on iron (Fe) availability; therefore plants regulate Fe homeostasis by tightly controlling its uptake and allocation. Fe, although abundant in soil, is not so readily available to plants in soils with high pH due to poor solubility (Colombo et al., 2014). Hence, plants employ different mechanisms for efficient acquisition of Fe from soil. To date, two mechanisms have been identified in higher plants, namely Strategy I or the reduction strategy and Strategy II or the chelation strategy (Kobayashi and Nishizawa, 2012; Connorton et al., 2017) for Fe acquisition.

Arabidopsis uses Strategy I mode to acquire Fe from soil (Kobayashi and Nishizawa, 2012). For Fe uptake, large amounts of coumarins, facilitated by PLEIOTROPIC DRUG RESISTANCE 9 (PDR9) (Fourcroy et al., 2014; Clemens and Weber, 2016) and protons, mediated by H^+^-ATPASE 2 (AHA2) (Santi and Schmidt, 2009) are pumped into the rhizosphere. These processes help to solubilize and mobilize the insoluble ferric Fe (Fe^3+^) in the rhizosphere (Chen et al., 2017; Jeong et al., 2017). Arabidopsis then reduces the soluble Fe^3+^ into ferrous Fe (Fe^2+^) by the action of FERRIC REDUCTASE OXIDASE 2 (FRO2) (Robinson et al., 1999) at the cell surface. And the IRON-REGULATED TRANSPORTER 1 (IRT1), a plasma membrane localized divalent cation transporter, then imports ferrous Fe from the extracellular space (Connolly et al., 2002; Vert et al., 2002).

Iron uptake and transport is coordinated by the actions of transcription factors. Several basic helix-loop-helix (bHLH) transcription factors are involved in orchestrating Fe transport and utilization. A subgroup of IIIa bHLH member, FER-LIKE IRON DEFICIENCY-INDUCED TRANSCRIPTION FACTOR (FIT) is involved in controlling the Fe uptake via regulating the expression of *IRT1* and *FRO2* (Colangelo and Guerinot, 2004; Jakoby et al., 2004; Yuan et al., 2005). Members of subgroup Ib of the bHLH proteins (bHLH38/39/100/101) redundantly interact with FIT and control the Fe uptake-associated genes (Wang et al., 2007, 2013; Yuan et al., 2008). Recent studies have revealed the upstream transcriptional regulation under Fe-starvation. The subgroup IVc bHLH factors (bHLH34/104/105/115), form heterodimers among themselves, directly regulate the expression of *Ib bHLH* genes and indirectly regulate the expression of the *FIT* (Zhang et al., 2015; Li et al., 2016; Liang et al., 2017). The IVc bHLH protein levels are post-translationally controlled by BRUTUS (BTS), a hemerythrin E3 ligase, via proteosomal degradation (Selote et al., 2015). BTS has been proposed to be involved in Fe sensing (Kobayashi and Nishizawa, 2015). BTS negatively regulates the Fe-starvation responses. Hindt et al. showed that the BTS paralogs, BTS LIKE1 (BTSL1) and BTS LIKE2 (BTSL2) act redundantly as negative regulators of the Fe starvation response (Hindt et al., 2017). Therefore, both positive and negative regulators coordinately fine tune the plant responses under the Fe starvation response.

To understand the optimal balance between positive and negative regulation, it is important to shed light on the signaling that is specific to each regulator (positive or negative). By modulating selective signaling branches we might be able to dissect the Fe starvation transcriptional network and the related complicated transcriptional machinery. Many molecules/metabolites such as sucrose, putrescine, nitric oxide (NO) and *S*-nitrosoglutathione (GSNO), and the hormones auxin and ethylene participate in the signaling process and positively regulate Fe-deficiency transcription (Chen et al., 2010; Lin et al., 2015; Lucena et al., 2015; Liu et al., 2016; Zhu et al., 2016; Kailasam et al., 2018); whereas the hormones, cytokinin, abscisic acid (ABA) and jasmonic acid (JA) act negatively on the network (Liu et al., 2016; Cui et al., 2018). A recent study by Garcia et al. (2018) discussed the different signaling modes, in the form LODIS (LOng Distance Iron Signal) or LODIS-derived and also via NO/GSNO, to the transcription factors. We previously undertook a chemical screening and dissected the Fe-signaling pathway using a small-molecule named R7 (Kailasam et al., 2018). R7 blocked the transfer of the Fe-deficiency generated signal from NO to the FIT by inhibiting the cellular levels of GSNO, a carrier of NO bio-activity, whose levels are critical for the activation of *FIT* expression. By using the small-molecule R7, we clarified the signaling pathway from NO (Kailasam et al., 2018).

Despite these findings, the identity of the signal that is transferred to transcription factors from NO is still unclear. Moreover, it is not clearly known whether the Fe-dependent signal is conveyed to the transcription factors through only one route or through many routes. With this focus, we used a chemical biology approach to further dissect the signaling routes of Fe starvation response. The chemical screening undertaken yielded two small-molecules named R3 and R6 (R denotes Repressor of *IRON-REGULATED TRANSPORTER 1*), whose actions during Fe-starvation are uncovered in this report. Small-molecule treatment resulted in severe Fe-dependent chlorosis and decreased Fe levels in shoots. R6 inhibited the expression of both *FIT* and *Ib bHLH* genes whereas R3 only inhibited *FIT* expression. Our finding clearly reveals that these small-molecules modulate Fe-deficiency by targeting specific signaling branches to central transcription factors, further suggesting that multiple routes are used for transferring the Fe-deficiency born signals to the central transcription factors in roots. Our work also highlights that small-molecules can be used to decode novel signaling pathways that modulate the transcription factors responsible for Fe-deficiency.

## Materials and Methods

### Plant Growth Conditions

*Arabidopsis thaliana* Col-0 and the reporter line *Pro_IRT1_:LUC* (Kailasam et al., 2018) were used. Seeds were surface-sterilized for 4 min in 70% ethanol and treated for 8 min with 1.2% sodium hypochlorite containing 0.02% SDS, finally washed several times in double-distilled H_2_O. Two-day-stratified seeds were grown on half-strength Murashige and Skoog (½MS) (Duchefa Biochemie) medium supplemented with 2.3 mM MES, 1% sucrose and 0.7% type A agar (Sigma-Aldrich) (pH 5.8). For Fe-sufficiency treatments [50 μM Fe(II)-EDTA], ½MS was used. For the Fe0 condition, Fe was omitted ½MS containing 0 μM Fe(II)-EDTA], whereas for the –Fe condition, 100 μM FerroZine was added to the Fe0 medium. For small molecule treatment, the indicated concentration was added in the medium, whereas in mock treatments dimethyl sulfoxide (DMSO) was added. All plants in this study were grown under a 16-h light/8-h dark photoperiod at 23°C.

### Small Molecule Screening

The small molecules R3 and R6 were isolated by screening DIVERSet library (ChemBridge, United States) for inhibition of *Pro_IRT1_:LUC* expression (Kailasam et al., 2018). Briefly, the DIVERSet library compounds were dissolved in DMSO and added a final concentration of 100 μM to 48-well plates containing –Fe medium. Two to three ½MS-grown-seedlings of 5 day old were transferred to the wells. Two days after treatment, plants were subjected to luminescence analysis. For luminescence assay, plants were submerged in 0.5 mM luciferin solution that contain 0.01% Triton X-100 and kept for 10 min in the dark. The luminescence was then captured by using the IVIS Lumina imaging system (Xenogen Corp., United States) with 1-min exposure times.

### Protein Isolation and Immunoblot

Total protein isolation and western blot analysis were conducted according to (Shin et al., 2013). Ten-day-old seedlings underwent a small-molecule treatment for 3 day before analysis. Small molecules were used at a final concentration of 50 μM. Total protein from roots was extracted by using protein extraction buffer: 125 mM Tris-HCL (pH 6.8), 15% glycerol, 5.5% SDS, 0.05% 2-mercaptoethanol, and Protease Inhibitor Cocktail (Roche). SDS-PAGE followed by western-blotting was performed. Blots were probed with an anti-IRT1 antibody (Shanmugam et al., 2011).

### Chlorophyll Estimation

Nine-day-old seedlings that have been grown on ½MS media were transferred onto ½MS (Fe50) or Fe0 media with 0 or 50 μM small molecules. After a 9-day treatment, the leaves were harvested and their fresh weight was measured. Total chlorophyll was extracted in 1.0 ml of 80% acetone at 4°C in the dark for 12–16 h until the leaves became white. The clear supernatant was then analyzed in a spectrophotometer (Power Wave XS; Bio-TEK) at 470, 646, and 663 nm spectra. The total chlorophyll content was calculated according to (Wellburn, 1994).

### Determination of Elemental Contents

Tissue elements were estimated by inductively coupled plasma-optical emission spectrometry (ICP-OES; OPTIMA 5300; Perkin-Elmer) as described (Shanmugam et al., 2011). Ten-day-old seedlings from ½MS plates were transferred to ½MS or Fe0 media containing 0 or 50 μM small molecules and grown for 10 days. Shoots were harvested and rinsed with 10 mM CaCl_2_ for 20 min. After washing with de-ionized water, shoots were dried at 70°C for 3 day, then digested with 1 ml 65% HNO_3_ (Merck, Tracepur) and 0.5 ml H_2_O_2_ (Merck, Suprapur). Digested samples were analyzed in ICP-OES for quantification.

### Quantitative Reverse Transcription-PCR

For gene expression analysis, 9-day-old seedlings from ½MS plates were transferred to ½MS or –Fe (½MS without Fe^2+^-EDTA and with 100 μM FerroZine) containing 0 or 25 μM small molecules. After 3 days of treatment, the roots were harvested. RNA isolation, complementary DNA (cDNA) synthesis and quantitative reverse transcription-PCR (qPCR) analysis were conducted according to manufactures protocol. In short, the total RNA was extracted by using Total RNA isolation kit (GeneDireX). The RNA samples were treated with gDNA wipeout RNase-free DNase (Qiagen) at 42°C for 2 min for genomic DNA contamination elimination. Approximately 1 μg of total RNA was used for first-strand cDNA synthesis by using a QuantiTect Reverse Transcription kit (Qiagen). 25 ng of RNA was subjected for quantitative PCR (qPCR) using Fast SYBR Green Master Mix (Applied Biosystems) in a 7500 Fast Real-Time PCR instrument (Applied Biosystems). Three biological replicates were used for the quantification of expression of each gene. Each biological replicate was analyzed in triplicate. Relative transcript levels were calculated by normalizing to *UBC21*. Expression was calculated by using the formula 2^-ΔCT^ (Schmittgen and Livak, 2008). The primers described (Zhang et al., 2015) were used for *bHLH100* and *bHLH101*. The primers described in (Shin et al., 2013) were used for *IRT1* and *UBC21*. The primers described in (Shanmugam et al., 2015) were used for *FRO2* and *FIT*. The primers described in (Kailasam et al., 2018) were used for *bHLH38* and *bHLH39*.

### Statistical Analysis

All statistical significance was determined using Student’s t test (*P* < 0.05) with SigmaPlot.

## Results

### Small Molecules R3 and R6 Block Fe-Deficiency-Induced IRT1 Expression

**FIGURE 1:**
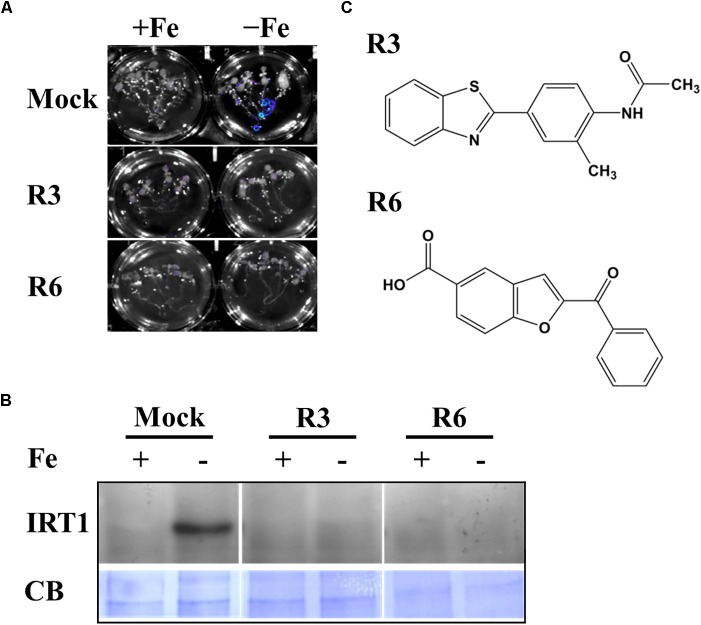
Small-molecules block Fe-deficiency response. **(A)** Small-molecules inhibit *Pro_IRT1_:LUC* expression. 6-days ½MS-grown plants were treated for 3 days under +Fe or -Fe media in the presence or absence of 50 μM indicated small-molecules. **(B)** IRT1 protein accumulation in response to small-molecules. The 10-days ½MS-grown plants were treated for 3 days under +Fe or -Fe in the presence or absence of small-molecules. IRT1 protein was detected in total protein extract of roots using an anti-IRT1 antibody. Samples were separated in the same gel. CB, coomassie blue stain. **(C)** The 2D structure of small-molecules. R3, N-[4-(1,3-benzothiazol-2-yl)-2-methylphenyl] acetamide; R6, 2-benzoyl-1-benzofuran-5-carboxylic acid.

We previously employed a chemical screen on *Pro_IRT1_:LUC* reporter lines and isolated a small-molecule named R7 (R denotes repressor of *IRON-REGULATED TRANSPORTER 1*) that represses the Fe-deficiency response in Arabidopsis (Kailasam et al., 2018). This screen yielded two more small molecules named R3 and R6. In this report, we analyzed the physiological and molecular responses of plants to understand the role of R3 and R6 in detail. R3 or R6 treatment inhibited the Fe-deficiency-inducible *Pro_IRT1_:LUC* expression (Figure 1A). Small-molecule treatment caused no luminescence in roots under Fe-deficient medium as compared to mock-treated that showed stronger luminescence. These results suggest that R3 and R6 may modulate endogenous *IRT1* expression.

First, to confirm that the small-molecule effect is not due to dysfunctional *Pro_IRT1_:LUC* under the treatment, we analyzed the endogenous IRT1 level under both Fe sufficient and deficient conditions (Figure 1B). As expected, the IRT1 protein was accumulated under Fe deficiency in mock treatment, whereas R3 or R6 treatment abolished the IRT1 accumulation. This indicates that R3 and R6 (Figure 1C) block the accumulation of Fe-deficiency-induced IRT1 protein.

### R3 and R6 Cause Severe Fe-Deficiency Chlorosis

**FIGURE 2:**
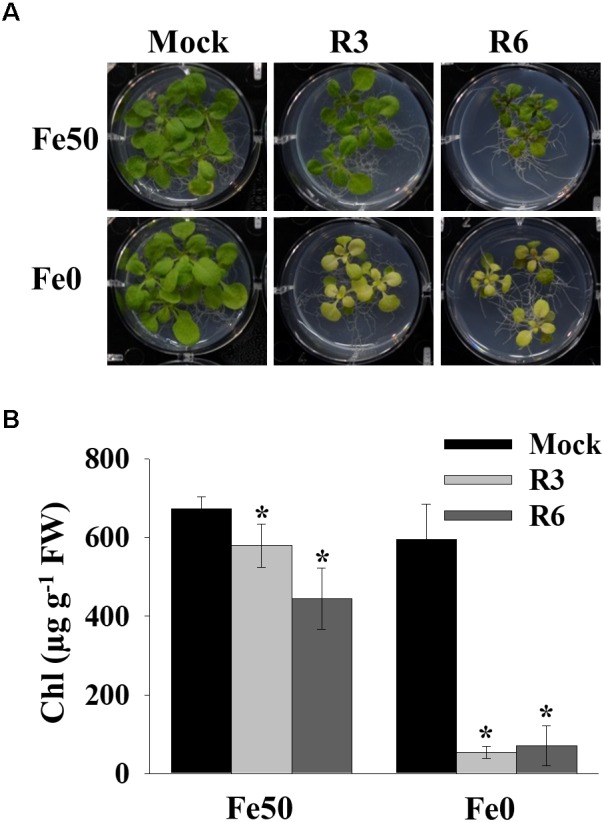
Small-molecules cause severe Fe-deficiency chlorosis. **(A)** Phenotype of plants with small-molecule treatments. 9-days ½MS-grown plants were transferred to Fe50 or Fe0 media for 9 days in the presence or absence of 50 μM indicated small-molecules. **(B)** Total chlorophyll content of plants under small-molecules treatment. Plants were treated as in **(A)**. Data are mean ± *SD* (*n* = 5). ^∗^, significant change vs. mock at *P* < 0.05 by Student’s *t* test. FW, fresh weight.

Iron deficiency in the environment causes chlorosis and affects the chlorophyll level in plants. To investigate the effect of small-molecule treatment on plant photosynthetic capacity under Fe starvation, phenotypic analysis was conducted (Figure 2). Compared to mock plants whose leaves were pale-green when grown under Fe-limited conditions, small molecule-treated plants were highly chlorotic (Figure 2A). We further measured the chlorophyll level in both Fe sufficient and deficient conditions. The small molecule treatment caused a decrease in levels of chlorophyll even under Fe-sufficiency and the levels were drastically reduced under Fe-limited conditions (Figure 2B). These data indicate that R3 and R6 perturb the physiological responses to Fe starvation.

### Small-Molecule Treatment Affects Metal Content

**FIGURE 3:**
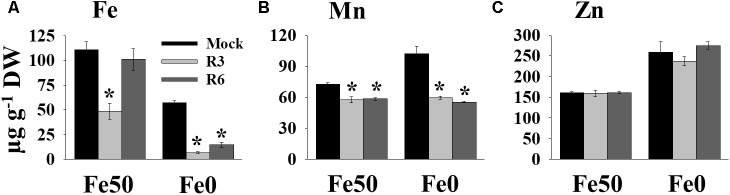
Small-molecules affect Fe levels in plants. Effect of small molecules on Fe **(A)**, Mn **(B)**, and Zn **(C)** contents in shoots. 10-days ½MS-grown plants were treated with or without 50 μM indicated small-molecules under Fe50 or Fe0 condition for 10 days. Levels of elements were measured by ICP-OES. Data are mean ±*SD* (*n* = 3). Significant differences compared with mock by Student’s *t* test: ^∗^, *P* < 0.05. DW, dry weight.

Perturbation in cellular levels of metals often results in chlorosis (Vert et al., 2002). R3 and R6 caused chlorosis; therefore, we next analyzed the cellular level of Fe in response to small molecule treatment (Figure 3). Under Fe sufficiency, R6 treatment did not alter the shoot Fe level, whereas R3 treatment led to a decrease in shoot Fe level (Figure 3A). In Fe-limited medium, the mock treatment showed reduced Fe levels, as expected. The small-molecule treatment caused a drastic reduction in the levels of shoot Fe under Fe-limited conditions. Since, IRT1 also transports manganese (Mn) and zinc (Zn), we then measured Mn and Zn levels. The Mn levels were significantly decreased in response to small molecule treatment under both Fe-sufficiency and -deficiency (Figure 3B). R3 or R6 treatment did not alter the Zn levels in shoots (Figure 3C). These data indicate that R3 and R6 treatment affect cellular metal contents, particularly Fe.

### Fe-Acquisition Genes Are Down Regulated in Small-Molecule Treatments

**FIGURE 4:**
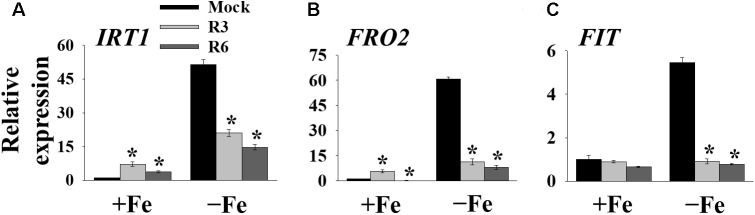
The expression of Fe-acquisition genes is inhibited by small-molecules. qPCR analysis of expression of *IRT1* **(A)**, *FRO2* **(B)**, and *FIT* **(C)** in roots. 9-days ½MS-grown plants were transferred to +Fe or -Fe in the presence or absence of 25 μM indicated small-molecules for 3 days. The expression of *UBC21* was used to normalize mRNA levels. The gene expression levels in mock +Fe were set to 1. Data are mean ± *SE* (*n* = 3). Significant differences compared with mock by Student’s *t* test: ^∗^, *P* < 0.05.

The above findings suggested that small-molecule treatment might impair the transcription of genes involved in Fe uptake. To test this, we measured the expression levels of Fe-uptake-associated genes, *IRT1*, *FRO2* and *FIT*, in response to small molecule treatment in roots. Loss-of-function mutants of these genes display a decrease in cellular Fe levels and chlorosis. We found that *IRT1*, *FRO2*, and *FIT* expression was induced 51.4-, 60.7- and 5.4-fold, respectively by Fe-deficiency in mock-treated plants (Figure 4). The small-molecule treatment strongly inhibited the transcripts of these genes under Fe-deficiency. These results indicate that R3 and R6 inhibit the molecular response to Fe-deficiency by affecting the central transcription factor.

### R3 and R6 Are Involved in Different Signaling Branches of Fe-Deficiency

**FIGURE 5:**
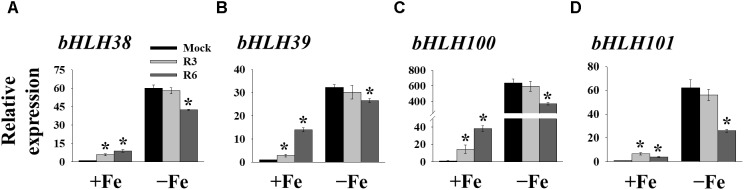
The expression of *Ib bHLH* genes is deregulated by R6 but not by R3. qPCR analysis of expression of *bHLH38* **(A)**, *bHLH39* **(B)**, *bHLH100* **(C)**, and *bHLH101* **(D)** in roots. 9-days ½MS-grown plants were treated for 3 days under +Fe or -Fe in the presence or absence of 25 μM indicated small-molecules. The expression of *UBC21* was used to normalize mRNA levels. The gene expression levels in mock +Fe were set to 1. Data are mean ± *SE* (*n* = 3). Significant differences vs. mock by Student’s *t* test: ^∗^, *P* < 0.05.

FIT forms a dimeric complex with members of the Ib bHLH factors (bHLH38/39/100/101) to regulate the expression of Fe-uptake genes, *IRT1* and *FRO2*. Fe-deficiency also induces the transcripts of *Ib bHLH* genes. Hence, we wondered whether small molecule treatment deregulates the expression of these genes as well or not. Expression analysis of *bHLH38/39/100/101* revealed that their transcripts are indeed induced in mock-treated plants under Fe-deficiency (Figure 5). Interestingly the R3 treatment did not influence the transcript levels of *Ib bHLH* genes under Fe-deficiency whereas R6 treatment inhibited the expression. The R6 inhibition level was 29.4, 17.6, 41.8, and 58.0% of the mock treatment for *bHLH38*, *bHLH39*, *bHLH100*, and *bHLH101*, respectively. These data indicate that R3 is not involved in the pathway for *Ib bHLH* gene expression whereas R6 is and further implies that small molecules R3 and R6 modulate the Fe-deficiency transcriptional networks selectively.

**FIGURE 6:**
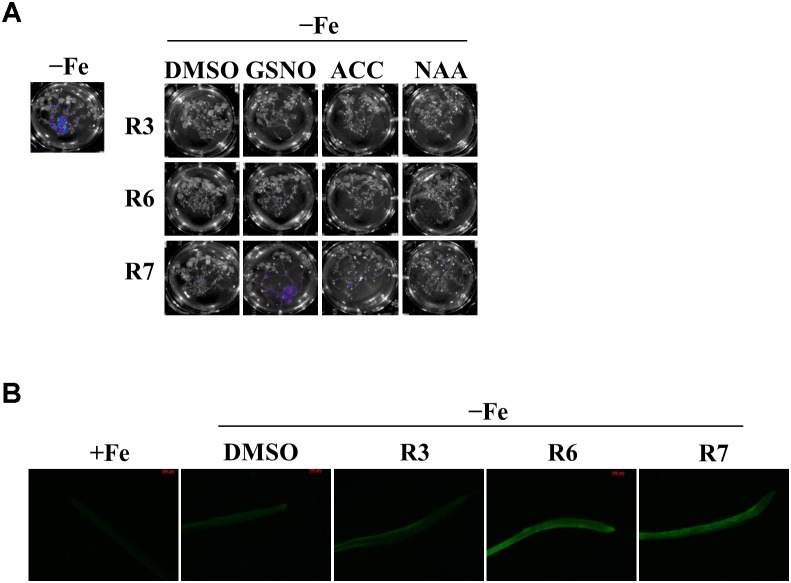
GSNO or ACC or NAA did not rescue the inhibitory effect of R3 or R6 on Fe-deficiency response. **(A)** Reverting small-molecule effect by GSNO, ACC, and NAA. 8-days ½MS grown plants were transferred to -Fe medium with 25 μM small-molecules in the presence or absence of 100 μM GSNO or 10 μM ACC or 0.2 μM NAA for 2-days. **(B)** NO level is not affected by R3 or R6 or R7. 8-days ½MS grown plants were transferred to -Fe with or without 50 μM R3 or R6 or R7 for 4-days. The NO was imaged in root tip using 5 μM DAF-FM DA dye.

The reduced expression of Fe-deficiency response transcription factors under small molecule treatment could be the result of defective signaling from plant hormones/metabolites. Auxin, ethylene, NO and GSNO act as positive regulators and exogenous applications of these substances are able to improve plant molecular response and fitness under Fe-starvation. Hence, we were interested in investigating whether providing these substances could alleviate the inhibitory effects caused by R3 or R6. We monitored the *Pro_IRT1_:LUC* expression under R3, R6, or R7, a small-molecule that blocks the signal from NO to FIT (Kailasam et al., 2018). *LUC* expression was not rescued under R3 or R6 treatment by providing any of the positive regulators [naphthaleneacetic acid (NAA) or 1-Aminocyclopropane-1-carboxylic acid (ACC) or GSNO] (Figure 6A). Under R7 treatment, supplying NAA or ACC did not rescue the *LUC* expression either, but supplying GSNO alleviated the R7 inhibition as demonstrated previously (Kailasam et al., 2018). Further we also measured the NO levels under these small-molecule treatments (Figure 6B). There was sufficient NO level, in fact higher, in roots under Fe deficiency upon treatment with any of the small-molecules. These data suggest that R3 and R6 act independently or downstream of these positive regulators.

### Plant Responses to Structural Derivatives of R3 and R6

**FIGURE 7:**
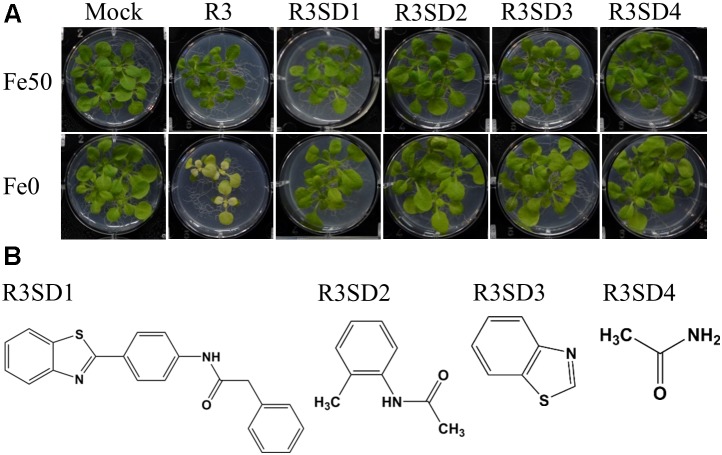
Effect of R3 structural derivatives. **(A)** The structural derivatives do not mimic the R3 effect. 9-days ½MS-grown plants were transferred to Fe50 or Fe0 medium for 9 days in the presence or absence of 50 μM small-molecules. **(B)** The 2D structure of R3 structural derivatives.

**FIGURE 8:**
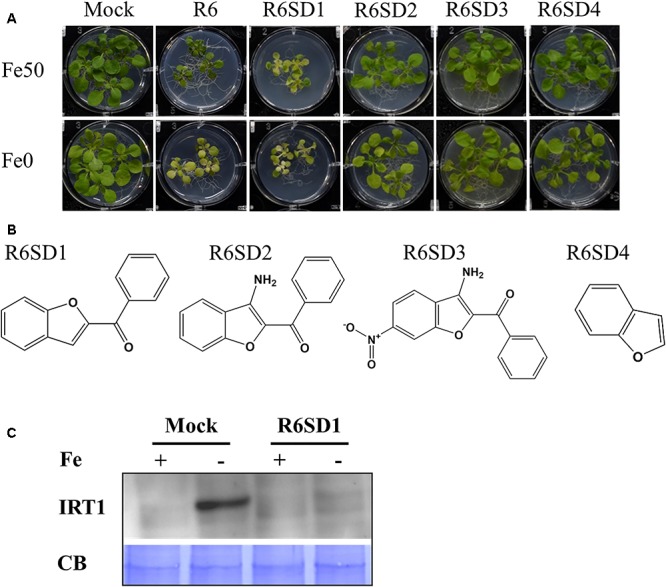
Effect of R6 structural derivatives. **(A)** The phenotype of plants under structural derivatives treatment. 9-days ½MS-grown plants were treated in Fe50 or Fe0 for 9 days in the presence or absence of 50 μM small-molecules. **(B)** The 2D structure of R6 structural derivatives. **(C)** IRT1 protein accumulation in roots under R6SD1 treatment. 10-days ½MS-grown plants were transferred to +Fe or -Fe with or without R6SD1 for 3 days. IRT1 protein was detected using an anti-IRT1 antibody. CB, coomassie blue stain.

Next, to get in-depth insight into the core motif that is required for the action of R3 and R6, structural analogs of the R3 and R6 were searched online in PubChem^1^ and ChemSpider^2^. We randomly selected some of the structural derivatives of R3 and R6 (Table 1), and assayed them. Our results showed that none of the four analogs of R3 assayed had any of the parent activity (Figure 7). They did not produce any observable phenotype under Fe limited conditions. In case of R6, one analog, R6SD1, mimicked the R6 activity; in fact it produced much stronger chlorosis and growth reduction than R6 under both Fe-sufficiency and deficiency (Figure 8). In addition, R6SD1 treatment diminished the IRT1 protein accumulation in roots under Fe-deficiency (Figure 8C). The structural analogs, therefore, may help to determine the active motif of the small molecule.

**Table 1:** Characteristics of small molecules.

Small molecule	IUPAC name	MW	Molecular formula	ChemSpider ID	PubCem CID
R3	N-[4-(1,3-benzothiazol-2-yl)-2-methylphenyl]acetamide	282.36	C_16_H_14_N_2_OS	349964	394824
R3SD1	N-[4-(1,3-benzothiazol-2-yl)phenyl]-2-phenylacetamide	344.43	C_21_H_16_N_2_OS	1146425	1370084
R3SD2	N-(2-methylphenyl)acetamide	149.19	C_9_H_11_NO	10298354	–
R3SD3	1,3-benzothiazole	135.18	C_7_H_5_NS	6952	7222
R3SD4	Acetamide	59.06	C_2_H_5_NO	173	178
R6	2-benzoyl-1-benzofuran-5-carboxylic acid	266.25	C_16_H_10_O_4_	6337783	8033570
R6SD1	1-benzofuran-2-yl(phenyl)methanone	222.24	C_15_H_10_O_2_	21133775	–
R6SD2	(3-amino-1-benzofuran-2-yl)-phenylmethanone	237.25	C_15_H_11_NO_2_	746595	854225
R6SD3	(3-amino-6-nitro-1-benzofuran-2-yl)-phenylmethanone	282.25	C_15_H_10_N_2_O_4_	4239080	5061996
R6SD4	1-benzofuran	118.13	C_8_H_6_O	8868	9223


## Discussion

Crop improvement toward fortification of Fe has great significance for human health as large populations depend on plant-feeds for dietary Fe. Enhancing Fe levels in plants is therefore useful. In order to achieve this, however; an adequate knowledge of Fe homeostasis is needed. Fe homeostasis in plants is controlled through at least five cellular processes: uptake systems, internal transport and distribution, utilization, storage, and finally the regulation (Connorton et al., 2017). Of these coordinated process, uptake is the most critical, that depends on soil pH, redox environment and interactions with other minerals (Colombo et al., 2014). To overcome this kind of environment and for efficient uptake, plants have evolved sophisticated mechanisms. Until now two systems for Fe-uptake, Strategy I, and Strategy II have been identified (Kobayashi and Nishizawa, 2012). Much meticulous work has helped to establish the Fe uptake and transport and the regulation process in Arabidopsis (Brumbarova et al., 2015; Curie and Mari, 2017). However, despite this knowledge, the precise sensing, both external and internal, and the associated signaling for Fe availability is still a poorly understood process.

Small-molecule-based chemical biology is an effective approach to dissect the nutrient-starvation response, especially signaling (Bonnot et al., 2016; Kailasam et al., 2018). In the current study, we investigated the role of two small-molecules, R3 and R6 in Fe deficiency response (Table 1). R3 and R6 blocked Fe-deficiency induced IRT1 expression (Figure 1). The induction of *IRT1* is important for coping with Fe-deficiency (Connolly et al., 2002; Vert et al., 2002). Physiological analysis revealed that R3 and R6 severely affected the chlorophyll levels in plants and imparted stronger chlorosis under Fe-limited conditions (Figure 2). This might be the result of reduced Fe levels under small molecule treatment (Figure 3A). Interestingly, under Fe sufficiency, R3-treated plants had a low Fe level with apparent chlorosis as well. By contrast, R6 treatment reduced the chlorophyll content, with no decrease in shoot Fe level under Fe sufficiency (Figure 2, 3A). Hence, total Fe level is not the only reason for chlorosis. The missing link between Fe content and chlorosis was also observed in the triple mutant *bhlh39bhlh100bhlh101*, which showed no defective total Fe level in shoots but strong chlorosis (Maurer et al., 2014). Treatment with R3 or R6 affected the Mn level (Figure 3B). Surprisingly the Zn level, whose level is subjected to increase under Fe-deficiency (Korshunova et al., 1999; Vert et al., 2002), is not affected under both Fe-sufficient and -deficient conditions (Figure 3C). One possibility for the unchanged Zn levels in shoots is higher translocation rate for Zn. Translocation of Fe, Mn and Zn is depends on the metal-chelating nicotianamine (NA)/citrate levels in the vasculature (Durrett et al., 2007; Schuler et al., 2012). Formation of NA-Zn complexes over Fe and Mn could be favored under conditions of limited Fe and the limiting levels of vasculature NA (Palmer et al., 2013). If R3 or R6 treatment brings down the levels of NA, then Zn would be the favorable substrate for the translocation. Other possibility might account for is that small-molecules may block the transport of metals in a selective manner.

Many genes are strongly induced in response to Fe-deficiency (Buckhout et al., 2009). Both R3 and R6 treatments inhibited the expression of Fe-uptake-associated genes *IRT1* and *FRO2* (Figure 4A,B). This inhibition is due to low transcript levels of central transcription factor *FIT* under Fe-deficiency (Figure 4C) upon small molecule treatment. FIT is the central modulator and is responsible for the activation of many Fe-deficiency-associated genes in root epidermal cells (Mai et al., 2016). We found that R6 downregulated the expression of *Ib bHLH* genes, FIT-partners under Fe-deficiency, whereas R3 did not (Figure 5). Our findings thus reveal that R3 and R6 may target the transcriptional response through distinct branches under Fe starvation.

The expression of the transcription factors responsible for Fe-deficiency is regulated by the upstream signaling molecules. Any defect in the levels or activity of these signaling-molecules causes decreased expression of transcription factors, and exogenous supply increases the transcription factor expression (Chen et al., 2010; Liu et al., 2016; Kailasam et al., 2018). Based on our data, none of the signaling-molecules (auxin/ethylene/GSNO) alleviated the inhibitory effects caused by R3 or R6 when they were supplied externally (Figure 6). NO levels were higher under R3 or R6 treatment than in the mock (Figure 6C). This supports the notion that R3 and R6 work downstream of auxin/ethylene/NO/GSNO, or alternatively that a novel pathway to the transcription factor exists independent of these hormones (Figure 4–6).

**FIGURE 9:**
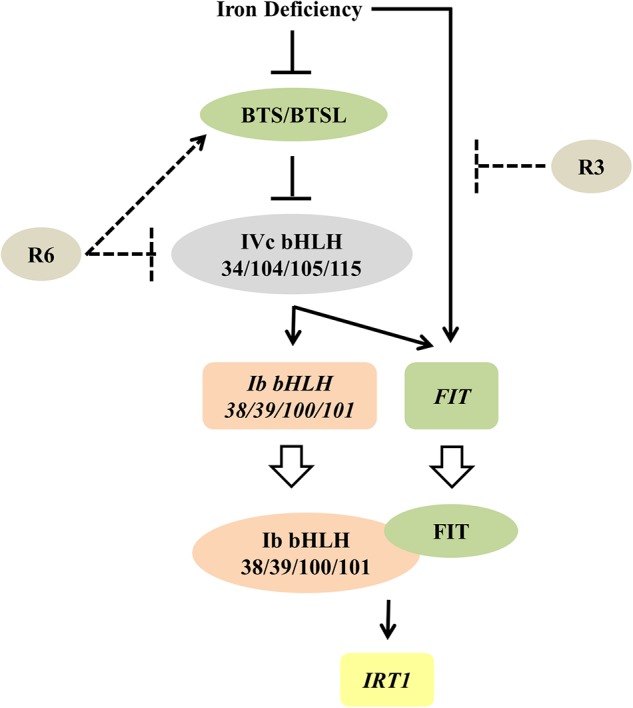
Proposed model for the possible action of R3 and R6 in Fe-deficiency transcriptional-network of Arabidopsis. Fe-status determines the BTS/BTSL stability/function. Under Fe deficiency, BTS is likely degraded, thereby allowing the accumulation of IVc bHLH proteins, which activates the transcription of *Ib bHLH* genes (directly) and *FIT* (indirectly). R6 inhibits the Fe-deficiency-stimulated expression of these IVc bHLH targets. R6 may block the IVc bHLH function by directly affecting the stability or indirectly promoting the degradation through BTS action. In addition to these signals, an unknown Fe-deficiency born signal directly activates the *FIT* transcription. FIT forms heterodimer with each member of Ib bHLH transcription factors and regulate the Fe-uptake gene *IRT1*. R3 most likely intercepts this unknown signal, thus repressing the *FIT* expression. Solid lines indicate data-supported information from previous studies. Open arrow indicates translation. Proteins are represented by colored-ovals and the genes are indicated by colored-boxes.

The observed decrease in expression of *FIT* and *Ib bHLH* transcription factors under R6 treatment (Figure 4, 5) might be due to a blocked signal passage from NO. It has been demonstrated that NO acts immediately upstream to these transcription factors but downstream of auxin (Chen et al., 2010; Garcia et al., 2010; Kailasam et al., 2018). Recently it has also been shown that IVc bHLH factors (bHLH34/104/105/115) directly control the *Ib bHLH* gene expression and indirectly control *FIT* (Zhang et al., 2015; Li et al., 2016; Liang et al., 2017). However, it is not clear whether or not IVc bHLH transcription factors work under NO. Our previous study showed that NO did not regulate the transcripts of *IVc bHLH* genes suggesting that control could be post-translational (Kailasam et al., 2018). One possibility that may account for the effect of R6 is that R6 may target these IVc bHLH proteins thereby reducing the expression of *Ib bHLH* and *FIT* genes (Figure 9). If this is so, it will be worth investigating how R6 regulates IVc factors.

Given that R3 treatment only affected the expression of *FIT* and not the *Ib bHLH* transcription factors (Figure 4, 5) and together with the data in Figure 6, it is highly likely that R3 targets the signaling route that is specific to FIT alone (Figure 9). Similar inhibition of the expression of Fe-homeostatic genes was also found with R7 treatment (Kailasam et al., 2018); however, R7 possibly interrupts the signaling pathway from NO to FIT. GSNO has been shown to be involved in mediating the signal from NO specifically to the FIT (Kailasam et al., 2018); however, the precise mechanism and the signal identity is unknown. As the external supply of GSNO did not rescue the R3 inhibition of *Pro_IRT1_:LUC* expression (Figure 6), R3 may interrupt the signal downstream of GSNO or target an independent unknown signaling pathway to FIT. We did not find any structural similarity between R3 and R7. This suggests the presence of multiple signal inputs for FIT, whose routes are selectively and independently targeted by the structurally less-related R3 and R7 compounds. Under Fe starvation, a wide range of chemical signals coordinate and trigger the transcriptional response (Liu et al., 2016). Some studies have suggested that cellular Fe, especially the levels in leaf vasculature itself act as a sensing/signaling component (Kumar et al., 2017; Garcia et al., 2018; Khan et al., 2018). Based on these findings, together with action of R3 and R7, it is clear that multilayered signaling networks exist. Importantly, there is lot of interconnection and feed-/forward-back between these signaling molecules, that influence each other, levels and activity under Fe-starvation (Garcia et al., 2011, 2018; Brumbarova et al., 2015; Liu et al., 2016). Therefore, further study of R3 may reveal the identity of a hidden unknown novel component that regulates the central transcription factor FIT.

Assaying the structural analogs of R3 did not help us to narrow down the active region of R3 and indicated that modifying the R3 parent compound will lead to loss of activity (Figure 7). R3 belongs to the benzothiazole class of compounds (Figure 1C and Table 1). Benzothiazole derived compounds are used in clinical studies and the benzothiazole moiety has been widely used as a template structure for the development of therapeutic agents (Ali and Siddiqui, 2013). However, the core benzothiazole (R3SD3) structure alone did not mimic the R3 effect and neither did the other R3 derivatives (Figure 7). Therefore, it seems that the parent structure of R3 itself is necessary for its activity. On the other hand, the structural derivatives of R6 provided some clues about the core motif required for the action of R6 (Figure 8). R6 is a benzofuran class compound. Benzofuran is an important pharmacophore and its derivatives are employed in medicinal chemistry for a wide range of drugs (Khanam and Shamsuzzaman, 2015). The benzofuran core (R6SD4) itself did not produce any observable phenotype under Fe-limited conditions. However, one structural derivative R6SD1 mimicked the R6 effect, in fact the phenotype caused more effect than R6 (Figure 8). The main difference between R6 and R6SD1 is that presence of a carboxylic moiety (-COOH) at the fifth position of the benzofuran unit. R6SD1 does not have a –COOH moiety. It should therefore be worthwhile studying the effect of the –COOH moiety in R6 on Fe-deficiency response. Further, characterizing many structural analogs of R3 and R6 might help us to better understand the core motif required for chemical activity, which will in turn benefit identification of its cellular targets, an important study.

## Conclusion

The data presented here strongly support the view that small molecules target signaling pathways in the Fe starvation response network and specifically modulate a particular pathway. This work also shows the usefulness of small-molecules in dissecting known signal transduction pathway(s). Furthermore, the selective inhibition of signaling pathways suggests the usefulness of R3 and R6 and chemical genetics *per se* to interpret networks and to identify new components in Fe-signaling. Based on our observations, the small-molecule R3 targets a novel unknown signaling pathway to the transcription factor FIT, whereas R6 may influence the IVc bHLH transcription factors under Fe-starvation (Figure 9). In summary, this study unraveled a new unknown Fe-signaling route and increases our understanding of plant Fe starvation signaling.

## Author Contributions

K-CY conceived the research. SK designed and performed the experiments. SK and K-CY wrote the manuscript. W-FC performed the chemical library screening and western-blot. All authors read and approved the manuscript.

## Conflict of Interest Statement

The authors declare that the research was conducted in the absence of any commercial or financial relationships that could be construed as a potential conflict of interest.

## Footnotes

1 https://pubchem.ncbi.nlm.nih.gov

2 http://www.chemspider.com
